# Sustained VWF‐ADAMTS‐13 axis imbalance and endotheliopathy in long COVID syndrome is related to immune dysfunction

**DOI:** 10.1111/jth.15830

**Published:** 2022-08-04

**Authors:** Helen Fogarty, Soracha E. Ward, Liam Townsend, Ellie Karampini, Stephanie Elliott, Niall Conlon, Jean Dunne, Rachel Kiersey, Aifric Naughton, Mary Gardiner, Mary Byrne, Colm Bergin, Jamie M. O'Sullivan, Ignacio Martin‐Loeches, Parthiban Nadarajan, Ciaran Bannan, Patrick W. Mallon, Gerard F. Curley, Roger J. S. Preston, Aisling M. Rehill, Ross I. Baker, Cliona Ni Cheallaigh, James S. O'Donnell, the Irish COVID‐19 Vasculopathy Study (iCVS) Investigators, Niamh O’Connell, Kevin Ryan, Dermot Kenny, Judicael Fazavana

**Affiliations:** ^1^ Irish Centre for Vascular Biology, School of Pharmacy and Biomolecular Sciences Royal College of Surgeons in Ireland Dublin Ireland; ^2^ Department of Infectious Diseases St James's Hospital Dublin Ireland; ^3^ Department of Clinical Medicine, School of Medicine, Trinity Translational Medicine Institute Trinity College Dublin Dublin Ireland; ^4^ Department of Immunology St James's Hospital Dublin Ireland; ^5^ National Coagulation Centre St James's Hospital Dublin Ireland; ^6^ Department of Intensive Care Medicine St James's Hospital Dublin Ireland; ^7^ Department of Respiratory Medicine St James's Hospital Dublin Ireland; ^8^ Centre for Experimental Pathogen Host Research University College Dublin Dublin Ireland; ^9^ St Vincent's University Hospital Dublin Ireland; ^10^ Department of Anaesthesia and Critical Care Royal College of Surgeons in Ireland Dublin Ireland; ^11^ National Children's Research Centre Our Lady's Children's Hospital Crumlin Dublin Ireland; ^12^ Western Australia Centre for Thrombosis and Haemostasis, Perth Blood Institute Murdoch University Perth Western Australia Australia; ^13^ Irish‐Australian Blood Collaborative (IABC) Network Dublin Ireland

**Keywords:** convalescent COVID‐19, endothelial cell activation, immune dysfunction, long COVID, Weibel Palade body exocytosis

## Abstract

**Background:**

Prolonged recovery is common after acute SARS‐CoV‐2 infection; however, the pathophysiological mechanisms underpinning Long COVID syndrome remain unknown. VWF/ADAMTS‐13 imbalance, dysregulated angiogenesis, and immunothrombosis are hallmarks of acute COVID‐19. We hypothesized that VWF/ADAMTS‐13 imbalance persists in convalescence together with endothelial cell (EC) activation and angiogenic disturbance. Additionally, we postulate that ongoing immune cell dysfunction may be linked to sustained EC and coagulation activation.

**Patients and methods:**

Fifty patients were reviewed at a minimum of 6 weeks following acute COVID‐19. ADAMTS‐13, Weibel Palade Body (WPB) proteins, and angiogenesis‐related proteins were assessed and clinical evaluation and immunophenotyping performed. Comparisons were made with healthy controls (*n* = 20) and acute COVID‐19 patients (*n* = 36).

**Results:**

ADAMTS‐13 levels were reduced (*p* = 0.009) and the VWF‐ADAMTS‐13 ratio was increased in convalescence (*p* = 0.0004). Levels of platelet factor 4 (PF4), a putative protector of VWF, were also elevated (*p* = 0.0001). A non‐significant increase in WPB proteins Angiopoietin‐2 (Ang‐2) and Osteoprotegerin (OPG) was observed in convalescent patients and WPB markers correlated with EC parameters. Enhanced expression of 21 angiogenesis‐related proteins was observed in convalescent COVID‐19. Finally, immunophenotyping revealed significantly elevated intermediate monocytes and activated CD4+ and CD8+ T cells in convalescence, which correlated with thrombin generation and endotheliopathy markers, respectively.

**Conclusion:**

Our data provide insights into sustained EC activation, dysregulated angiogenesis, and VWF/ADAMTS‐13 axis imbalance in convalescent COVID‐19. In keeping with the pivotal role of immunothrombosis in acute COVID‐19, our findings support the hypothesis that abnormal T cell and monocyte populations may be important in the context of persistent EC activation and hemostatic dysfunction during convalescence.

**Essentials**

- Reduced plasma ADAMTS‐13 levels and an increased VWF/ADAMTS13  ratio are common in convalescentCOVID‐19 patients.
- Levels of Platelet Factor 4, a putative VWF protector, are elevated in convalescent COVID‐19 patients compared to healthy controls.
- Elevated levels of pro‐angiogenic proteins, including Weibel Palade Body constituents Angiopoietin‐2 and Osteoprotegerin, are observed in a subset of patients.
- Intermediate monocytes and activated CD4+ and CD8+ T cell subsets are increased in convalescent COVID‐19 and correlate with thrombin generation and endotheliopathy markers, respectively.

## 1 INTRODUCTION

Prolonged functional impairment following COVID‐19 disease, referred to as Long COVID or post‐acute COVID‐19 syndrome (PACS), is estimated to occur in 30%–40% of infected individuals.^1^, ^2^ Typical features include fatigue, breathlessness, and reduced exercise tolerance. However, the clinical phenotype is heterogeneous and proposed definitions vary.^3^, ^4^ Despite the global morbidity associated with Long COVID, the underlying pathogenic mechanisms are poorly understood. However, recent studies have demonstrated that, similar to acute illness, persistent coagulation activation is common in convalescent COVID‐19 patients.^5^, ^6^, ^7^, ^8^, ^9^ In particular, several groups have reported sustained increases in endogenous thrombin potential and D‐dimer levels in patients between 4 and 12 months following acute illness.^6^, ^7^, ^9^ Ongoing hemostatic dysfunction was more common in patients who required intensive care support during their acute COVID‐19 illness and in patients aged >50 years.^6^, ^7^ Interestingly, however, prolonged increased D‐dimer levels and coagulation activation were also seen in some patients who had mild COVID‐19 and were managed exclusively as out‐patients.^6^, ^7^

Post mortem studies have highlighted that acute SARS‐CoV‐2 infection is associated with specific deleterious effects on endothelial cells (EC).^10^, ^11^ Features include loss of normal tight EC junctions leading to increased EC permeability, enhanced EC apoptosis, and abnormal angiogenesis.^11^, ^12^, ^13^, ^14^ In keeping with the concept that SARS‐CoV‐2 infection is associated with marked EC activation, plasma levels of von Willebrand factor antigen (VWF:Ag), VWF propeptide (VWFpp), and Angiopoietin‐2 (Ang‐2) are all significantly increased and correlate with clinical severity.^15^, ^16^, ^17^ In addition, reduced plasma ADAMTS‐13 levels and abnormal ultra‐large VWF multimers have been observed in acute COVID‐19.^18^, ^19^ Collectively, these data suggest that acute EC activation and dysregulation of the normal VWF/ADAMTS‐13 axis play important roles in the pathobiology underlying pulmonary immunothrombosis and microvascular occlusion in acute COVID‐19. Interestingly, recent studies have further reported that plasma VWF:Ag, VWFpp, and factor VIII (FVIII) levels all remain significantly elevated after 3 months in convalescent COVID‐19 patients compared to healthy controls.^6^ Based on these data, roles for sustained EC activation in the pathogenesis of persistent hemostatic dysfunction and Long COVID symptomatology have been proposed. In this study, we sought to further investigate the nature of ongoing endotheliopathy and VWF–ADAMTS‐13 axis dysfunction in convalescent COVID‐19.

## 2 METHODS

Sequential convalescent COVID‐19 patients (*n* = 50) who had blood samples taken at time of outpatient review in the Long COVID clinic at St James's Hospital (SJH) between May and September 2020, were included. Acute COVID‐19 patients from Beaumont Hospital and SJH (*n* = 36) and a control group of age‐ and sex‐matched asymptomatic controls (*n* = 20) were also recruited. Informed written consent was obtained from participants and ethical approval granted by the Hospital Research Ethics Committee. All acute (*n* = 36) and convalescent COVID‐19 patients (*n* = 50) had coagulation samples (3.2% sodium citrate tubes) taken. Samples for immunophenotyping were also taken from a subset of patients (*n* = 37 convalescent and *n* = 32 acute COVID‐19 patients). Levels of VWF:Ag, VWFpp, interleukin‐6 (IL‐6), and soluble thrombomodulin (sTM) were quantified as before.^6^, ^20^ Factor VIII activity was measured by chromogenic assay (FVIII:C) in the National Coagulation Centre reference laboratory at SJH. Thrombin generation was performed in a Fluouroskan Ascent Fluorometer with Thrombinoscope software (Stago) using PPP Low reagent (1 pM tissue factor, 4 mM phospholipids) as before.^6^, ^21^ Characterization of peripheral blood monocyte and T lymphocyte populations by flow cytometry were performed as previously described.^22^ Plasma ADAMTS‐13 (R&D Systems #DY4245‐05), platelet factor 4 (PF4; R&D Systems #DY795), Ang‐2 (R&D Systems #DY623), and Osteoprotegerin (OPG; R&D Systems #DY805) levels were measured using commercial ELISAs. Angiogenesis profiles were assessed by membrane‐based antibody array according to the manufacturers' instructions (R&D Systems #ARY007). Clinical assessment included: chest x‐ray and 6‐min walk test (6MWT) measuring distance covered and lowest arterial oxygen saturation. Statistical analyses were performed using the Mann–Whitney *U* tests, Kruskal‐Wallis tests, and the Spearman rank correlation test in GraphPad Prism 9.0 (GraphPad Software) with a *p*‐value of <0.05 considered statistically significant.

## 3 RESULTS AND DISCUSSION

Convalescent COVID‐19 patients (*n* = 50, 60% male, median age 50 [interquartile range (IQR) 36–63] years) were assessed at a minimum of 6 weeks (median 68 [IQR 61–72] days), following either symptom resolution or hospital discharge, in keeping with previous definitions of Long COVID (Table 1).^1^ The majority of patients (37/50, 74%) required hospitalization, while just over one quarter (13/50, 26%) managed their acute illness at home. Comorbidities were identified in 31/50 (62%) patients (Table 1). Acute hospitalized COVID‐19 patients received weight‐ and renally adjusted low molecular weight heparin prophylaxis whereas non‐hospitalized and convalescent patients did not.

**TABLE 1:** Demographic and laboratory parameters of acute and convalescent COVID‐19 cohorts

Parameters	Normal range	Convalescent COVID‐19 (*n* = 50)	Acute COVID‐19 (*n* = 36)
Demographic data			
Age, median (IQR)		50 (27)	62 (16)
Male – *n* (%)		30 (60)	27 (75)
BMI, median (IQR)		28 (7)	30 (9)
Comorbidity count, median (IQR)		1 (3)	3 (2)
Hospitalization – *n* (%)		37 (74)	36 (100)
ICU – *n* (%)		8 (16)	18 (50)
Laboratory parameters at time of blood sampling – median (IQR)			
Leukocytes (×10^9^/L)	4–11	6.3 (1.7)	7.8 (5.3)
Lymphocytes (×10^9^/L)	1.5–3.5	1.9 (0.7)	1.1 (0.7)
Neutrophils (×10^9^/L)	2–7.5	3.3 (1.2)	5.9 (5.4)
Platelets (×10^9^/L)	140–450	272 (60)	276 (145)
Creatinine (μmol/L)	45–84	73 (26)	72 (34)
Fibrinogen (g/L)	1.9–3.5	2.9 (0.8)	4.8 (2.8)
D‐dimer (ng/ml)	0–500	377 (251)	980 (1619)
CRP (mg/ml)	0–5	1.1 (1)	110 (173)
IL‐6 (pg/ml)	0–7.26	1.5 (3.6)	76.6 (144.4)
Clinical parameters Convalescent COVID‐19 only – median (IQR) unless otherwise stated			
Time to follow‐up (days)		68 (11)	NA
6MWT distance (m)	400–700	430 (160)	NA
Lowest desaturation (%)		95 (2)	NA
Abnormal chest X‐ray – *n* (%)		3 (6)	NA

Abbreviations: 6MWT, 6‐minute walking test; BMI, body mass index; COVID‐19, coronavirus disease 2019; CRP, C‐reactive protein; ICU, intensive care unit; IL, interleukin; IQR, interquartile range.

**FIGURE 1:**
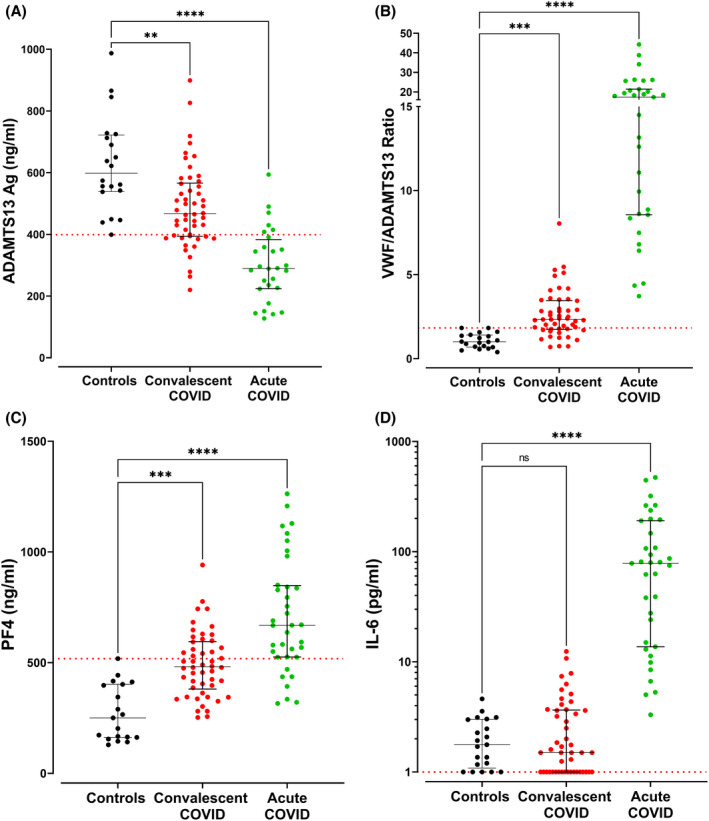
Comparisons are shown among acute COVID‐19 patients (*n* = 36), convalescent COVID‐19 patients (*n* = 50), and healthy controls (*n* = 20) including: (A) plasma ADAMTS‐13:Ag levels, (B) plasma VWF/ADAMTS‐13 ratios, (C) plasma Platelet Factor 4 (PF4) levels, and (D) plasma Interleukin‐6 (IL‐6) levels. Dotted red lines denote the lower limit of the local reference range for plasma ADAMTS‐13:Ag and IL‐6 levels and the upper limit of the local reference range for the VWF/ADAMTS‐13 ratio and PF4 levels. Data are presented as median and the interquartile range. Comparisons between groups were assessed using the Kruskal‐Wallis test (ns = not significant, **p <0 .01,***p < 0.001, ****p < 0.0001).

In keeping with previous studies, we confirmed significant increases in plasma VWF:Ag levels (Figure S1) and concurrent reductions in plasma ADAMTS‐13 levels (Figure 1A) in our acute COVID‐19 subgroup. As a result, the VWF‐ADAMTS‐13 ratio was markedly elevated >10 fold in acute COVID‐19 patients compared to controls (Figure 1B). In patients with convalescent COVID‐19, we observed that plasma ADAMTS‐13 levels were significantly reduced compared to controls (median 598 ng/ml vs. 630 ng/ml, *p* = 0.009; Figure 1A). Marked inter‐individual variation was apparent, with ADAMTS‐13 levels ranging from 220 ng/ml to 900 ng/ml in the convalescent cohort. Pertinently, plasma ADAMTS‐13 levels below the lower limit of our local normal reference range (399 ng/ml) were seen in 15/50 (30%) convalescent COVID‐19 patients, with a median ADAMTS‐13 level of 384 ng/ml in this subgroup. ADAMTS‐13 levels were significantly lower in convalescent COVID‐19 patients who had required hospitalization compared to those managed entirely as outpatients (*p* = 0.04; data not shown). The absolute reduction in ADAMTS‐13 levels in convalescent COVID‐19 patients was significantly less marked than that seen in patients with acute COVID‐19 (Figure 1A). Importantly, however, the reduction in ADAMTS‐13 levels coupled with elevated plasma VWF:Ag levels, meant that the VWF‐ADAMTS‐13 ratio remained significantly elevated in convalescent COVID‐19 patients compared to controls (median 2.3 vs. 1.0, *p* = .0004; Figure 1B). In fact, 35/50 (70%) convalescent COVID‐19 patients studied still had VWF‐ADAMTS‐13 ratios above the upper limit of the normal reference range (1.8). On univariate analysis, the VWF‐ADAMTS‐13 ratio was significantly higher in patients who had required hospitalization, those with ≥2 comorbidities, and those with reduced exercise tolerance (6MWT below the median distance, 430 m; data not shown). Interestingly, Prasannan et al. recently reported similar VWF/ADAMTS‐13 ratios in a convalescent COVID‐19 cohort using an ADAMTS‐13 FRETS assay, in which VWF/ADAMTS‐13 ratios >1.5 were noted in 28% and were associated with impaired exercise tolerance.^23^

In addition to the abnormal VWF/ADAMTS‐13 ratio in acute COVID‐19, previous studies have reported significant increases in a number of putative VWF protectors, including PF4 and IL‐6, which can regulate VWF cleavage by ADAMTS‐13.^18^, ^24^, ^25^ We observed that PF4 levels remained significantly increased in convalescent COVID‐19 compared to controls (median 481 pg/ml vs. 250 pg/ml, *p* =0.0001; Figure 1C). Notably, 23/50 (46%) of the convalescent COVID‐19 cohort had persistent PF4 levels >500 ng/ml, levels similar to those seen in acute COVID‐19. While it has been shown that PF4 binds to VWF and PF4–VWF complexes exist in thrombotic thrombocytopenic purpura,^25^ further studies are needed to determine the biological mechanisms underlying this sustained increase in plasma PF4 levels and whether similar PF4–VWF complexes exist in convalescent COVID‐19. Finally, in contrast to the PF4 findings, we observed that plasma IL‐6 levels had returned to within the normal range in the majority (46/50, 92%) of convalescent COVID‐19 patients (Figure 1D).

**FIGURE 2:**
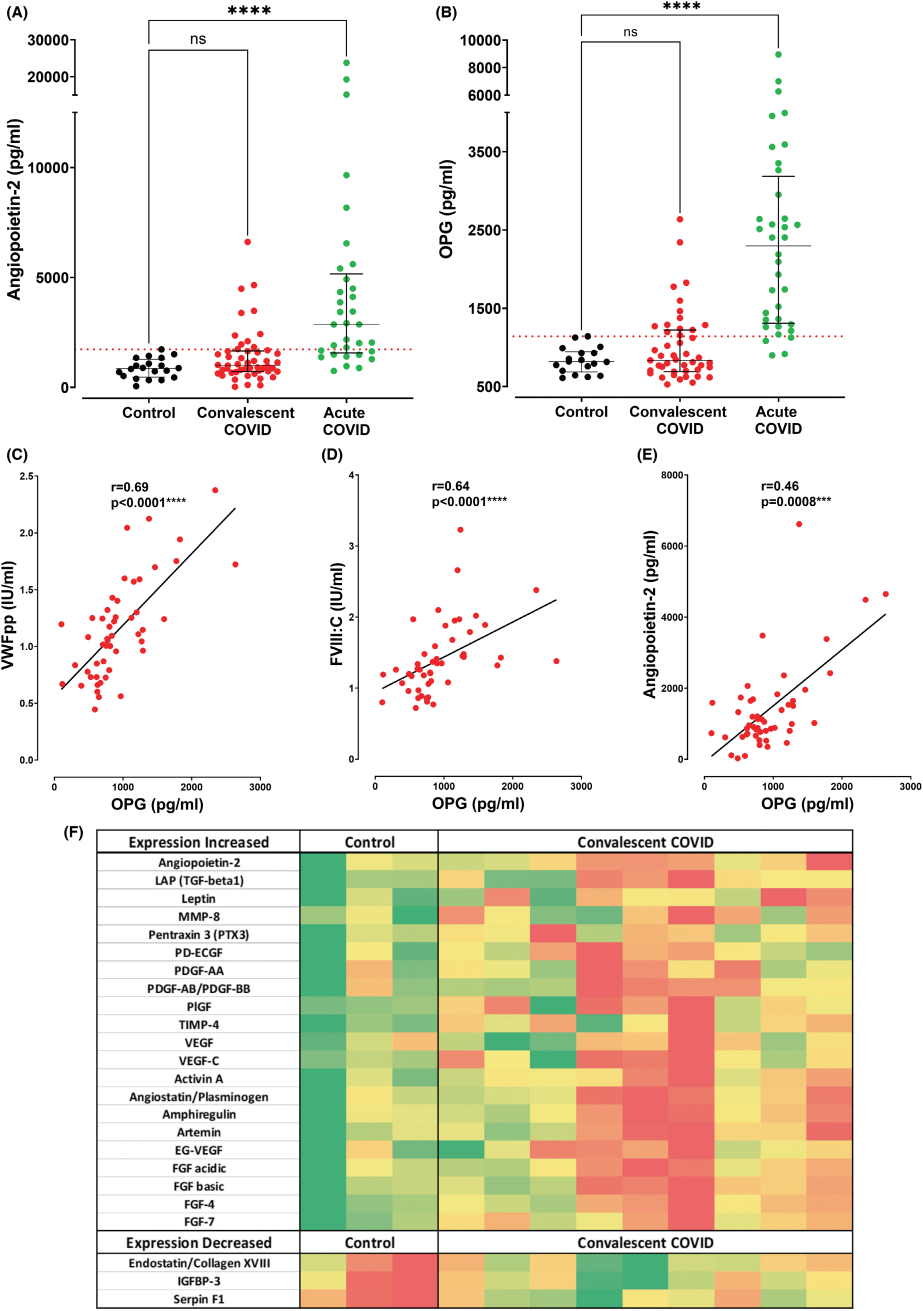
Comparisons are shown between acute COVID‐19 patients (*n* = 36), convalescent COVID‐19 patients (*n* = 50), and healthy controls (*n* = 20) including: (A) plasma Angiopoietin‐2 (Ang‐2) levels, and (B) plasma Osteoprotegerin (OPG) levels. Dotted red lines denote the upper limit of the local reference range for plasma Ang‐2 and OPG. Data are presented as median and the interquartile range. Comparisons between groups were assessed using the Kruskal‐Wallis test. Correlations are shown between plasma levels of OPG and endothelial cell activation parameters including: (C) von Willebrand factor propeptide (VWFpp), (D) Factor VIII:C, and (E) Angiopoietin‐2. Correlations were evaluated using the Spearman rank correlation test. (F) Heatmap visualization indicating angiogenesis‐related protein expression detected in each subject (columns) for each protein (rows), comparing a subset of convalescent COVID‐19 patients (*n* = 9, 6/9 males) and healthy controls (*n* = 3, 2/3 males). This subset of convalescent patients and controls were selected to match the age and sex profile of the overall convalescent cohort. Protein levels were measured via membrane‐based antibody array and data are represented by mean pixel intensity with red indicating higher and green indicating lower levels of the protein of interest. Comparisons between groups were assessed using the Mann–Whitney *U* test.(ns = not significant, ***p <0 .001, ****p <0 .0001).

The increases in plasma VWF:Ag, VWFpp, and FVIII:C levels seen in convalescent COVID‐19 suggest ongoing EC activation and Weibel Palade Body (WPB) exocytosis. Importantly WPB also store other important pro‐inflammatory and pro‐angiogenic molecules, including Ang‐2 and OPG.^26^, ^27^ To further investigate the hypothesis that sustained EC activation may be associated with ongoing exocytosis of WPB constituents in convalescent COVID‐19, we next investigated Ang‐2 and OPG levels. Overall, plasma Ang‐2 levels were non‐significantly increased in the convalescent cohort compared to healthy controls (median 1009 pg/ml vs. 859 pg/ml, *p* = .23; Figure 2A). However, significant inter‐patient variation was observed, with plasma Ang‐2 levels above the local normal range observed in 22% (11/50) of convalescent COVID‐19 patients (Figure 2A). Similarly, marked inter‐individual variability in plasma OPG levels was also seen in the convalescent COVID‐19 cohort (Figure 2B). Overall, there was a non‐significant increase in convalescent OPG levels compared to controls (median 835 pg/ml vs. 818 pg/ml, *p* = 0.9; Figure 2B). However, 14/50 (28%) of convalescent COVID‐19 patients had plasma OPG levels above the upper limit of the normal healthy controls. Consistent with the concept of ongoing endotheliopathy and WPB exocytosis in patients with convalescent COVID‐19, significant correlations were observed among OPG, VWFpp, FVIII, and Ang‐2 (Figures 2C–E).

In an autopsy study of fatal acute COVID‐19, Ackermann et al. reported significantly increased new vessel growth, predominantly through a mechanism of intussusceptive angiogenesis in the pulmonary vasculature.^11^ While data specifically on intussusceptive angiogenesis in acute COVID‐19 are limited, a larger study of 144 autopsy samples from fatal COVID‐19 similarly identified abnormal angiogenesis in multiple organ sites, with 139 angiogenesis‐related proteins being significantly dysregulated, compared to 74 control tissues from non‐COVID patients.^28^ Subsequent studies have found circulating pro‐angiogenic factors (including VEGF‐A, PDGF‐AA and PDGF‐AB/BB) were significantly elevated in acute COVID‐19 and correlated with disease severity.^29^ Because VWF, Ang‐2, and OPG can influence EC angiogenesis, we next performed a pilot study to assess a panel of 24 angiogenesis‐related proteins in a subset (*n* = 9) of our convalescent COVID‐19 patients and healthy controls (*n* = 3). Significant inter‐patient heterogeneity was again seen (Figure 2F). Overall, significantly (*p* <0 .05) enhanced expression of 21 angiogenesis‐related proteins was observed in the convalescent COVID‐19 subgroup compared to controls (Figure 2F). In keeping with previous studies in acute COVID‐19, elevated pro‐angiogenic markers included Ang‐2, as well as members of the VEGF, PDGF, placental growth factor (PGF), and fibroblast growth factor (FGF) families.^17^, ^29^ Conversely, plasma levels of endostatin and serpin F1 (both inhibitors of angiogenesis) were significantly reduced in convalescent COVID‐19 (Figure 2F). These preliminary data can serve as a platform for future larger studies exploring the concept that dysregulated angiogenesis is not only a feature of acute COVID‐19, but may also be sustained during convalescence in a subset of patients.

**FIGURE 3:**
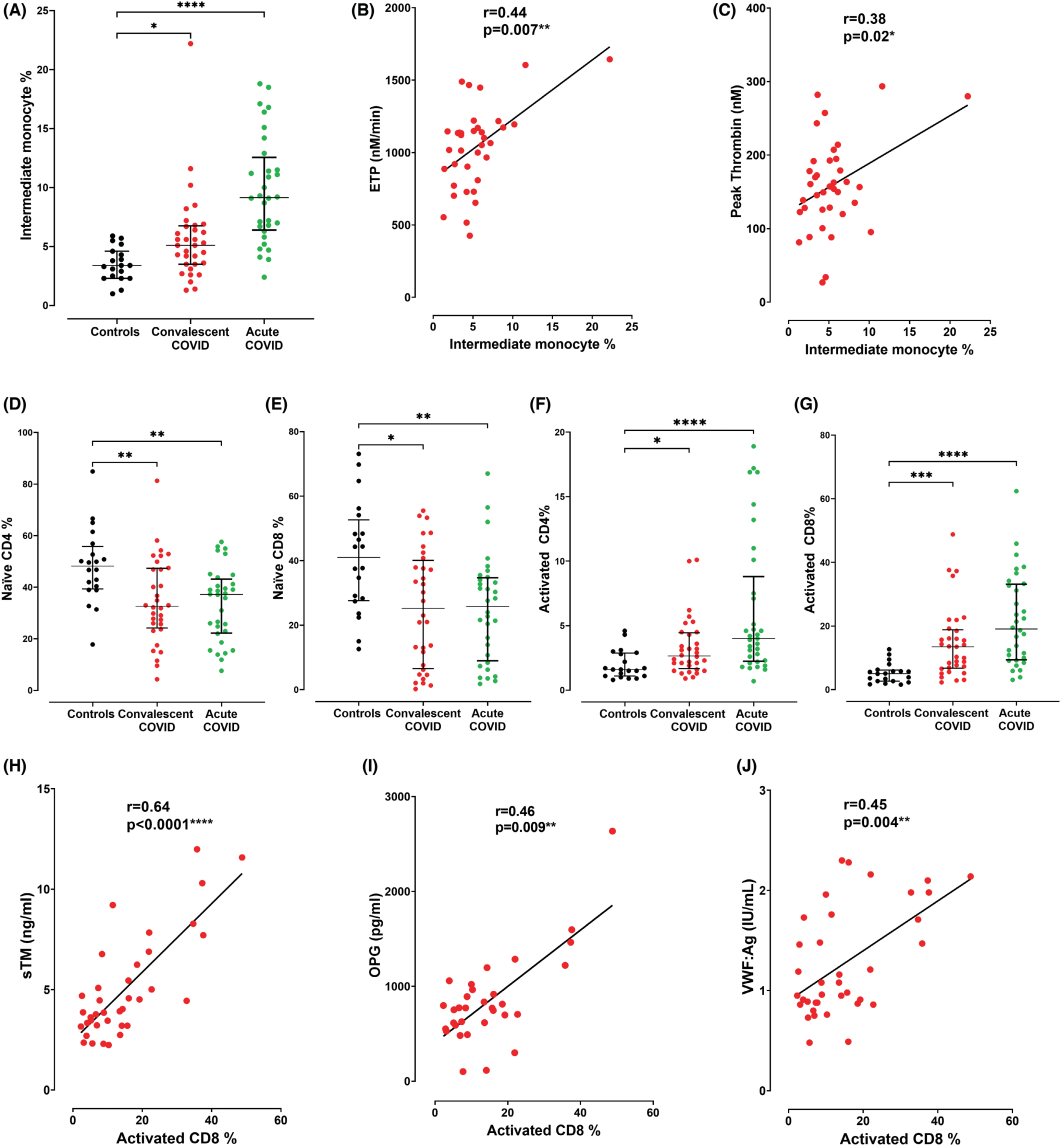
(A) Intermediate monocytes in peripheral blood were assessed using flow cytometry in convalescent COVID‐19 patients (*n* = 37), acute COVID‐19 patients (*n* = 32), and healthy controls (*n* = 20). Correlations between intermediate monocytes and (B) endogenous thrombin potential (ETP) and (C) peak thrombin generation, respectively. T lymphocyte subsets in peripheral blood were assessed using flow cytometry in convalescent COVID‐19 patients (*n* = 37), acute COVID‐19 patients (*n* = 32), and healthy controls (*n* = 20) including percentage of (D) naïve CD4, (E) naïve CD8, (F) activated CD4, and (G) activated CD8 positive T cells. Correlations are shown between activated CD8+ T cells and plasma levels of (H) soluble thrombomodulin (sTM), (I) Osteoprotegerin (OPG), and (J) VWF:Ag. Data are presented as median and the interquartile range. Comparisons between groups were assessed by the Mann–Whitney *U* test. Correlations were evaluated using the Spearman rank correlation test (ns = not significant, *p < 0.05, **p < 0.01, ***p < 0.001, ****p < 0.0001).

The molecular mechanisms responsible for persistent hemostatic abnormalities in convalescent COVID‐19 have not been defined. However, recent studies have described persistent immune profile abnormalities in convalescent COVID‐19 patients, including abnormal monocyte profiles and T cell activation.^22^ We hypothesized that sustained inflammatory cell abnormalities may be important in triggering ongoing thrombin generation and/or endotheliopathy. Consistent with previous reports, immunophenotyping revealed increased HLA‐DR + CD14 + CD16+ intermediate monocytes in convalescent (*n* = 37) and acute COVID‐19 patients (*n* = 32) compared with controls (*n* = 20; Figure 3A), whereas classical and non‐classical monocyte profiles were unchanged in convalescence (Figure S1). We and others have previously reported sustained increases in endogenous thrombin potential (ETP) and peak thrombin generation in convalescent COVID‐19 patients compared to controls.^6^ Interestingly, intermediate monocyte percentage correlated significantly (*p* = 0.007) with ETP and peak thrombin (*p* = 0.02) in our patient cohort (Figure 3B,C; Table S1). This is noteworthy as tissue factor induction on intermediate monocytes has been shown to contribute to platelet activation in acute COVID‐19.^30^ Finally, similar to previous studies, we observed significant reductions in naïve CD4+ and CD8+ T cell subsets in both convalescent (*n* = 37) and acute COVID‐19 patients (*n* = 32) compared to controls (*n* = 20; Figure 3D,E) with concomitant elevations in activated CD4+ and CD8+ T cell subsets (Figure 3F,G). Furthermore, activated CD4+ and CD8+ T cells correlated positively with EC and WPB parameters, including plasma soluble thrombomodulin (sTM), OPG and VWF:Ag levels (Figure 3H–J) whereas naïve CD4+ and CD8+ T cells correlated inversely with these markers (Table S2). Importantly, no significant associations were noted between (i) intermediate monocytes and EC activation markers or (ii) T cell activation subsets and thrombin generation parameters (Tables S1 and S2).

In conclusion, our data provide novel insights into the nature of sustained EC activation, WPB exocytosis, and VWF/ADAMTS‐13 axis imbalance in convalescent COVID‐19. In keeping with the pivotal role of immunothrombosis in acute COVID‐19, our findings support the hypothesis that abnormal T cell and monocyte profiles may be important in the context of persistent EC activation and ongoing hemostatic dysfunction during convalescence. Further studies will be required to determine how this prolonged EC and immune activation, together with dysregulated angiogenesis in convalescent patients, may contribute to functional impairment post‐COVID‐19.

## AUTHOR CONTRIBUTIONS

HF, SEW, LT, EK, SE, NC, CB, CNC, JOS, and JOD were responsible for conception, patient enrollment, data collection, and interpretation. All authors contributed to literature review, final draft writing, and critical revision. All the authors have participated sufficiently in this work, take public responsibility for the content, and have made substantial contributions to this research.

## CONFLICT OF INTEREST

J.S.O'D. has served on the speaker's bureau for Baxter, Bayer, Novo Nordisk, Sobi, Boehringer Ingelheim, Leo Pharma, Takeda, and Octapharma. He has also served on the advisory boards of Baxter, Sobi, Bayer, Octapharma CSL Behring, Daiichi Sankyo, Boehringer Ingelheim, Takeda, and Pfizer. J.S.O'D. has also received research grant funding awards from 3M, Baxter, Bayer, Pfizer, Shire, Takeda, and Novo Nordisk. The remaining authors have no conflicts of interest to declare.

## Supporting information


Figure S1

Tables S1–S2

Data S1

## APPENDIX 1

Additional investigators of the Irish COVID‐19 Vasculopathy Study (ICVS): Niamh O'Connell (National Coagulation Centre, St James's Hospital, Dublin), Kevin Ryan (National Coagulation Centre, St James's Hospital, Dublin), Dermot Kenny (Irish Centre for Vascular Biology, Royal College of Surgeons in Ireland), Judicael Fazavana (Irish Centre for Vascular Biology, Royal College of Surgeons in Ireland).
