# Supplementary figures and images for: Sustained VWF‐ADAMTS‐13 axis imbalance and endotheliopathy in long COVID syndrome is related to immune dysfunction
Source: J Thromb Haemost. 2022 Aug 4;20(10):2429–38. doi: 10.1111/jth.15830 (PMC9349977; doi:10.1111/jth.15830)

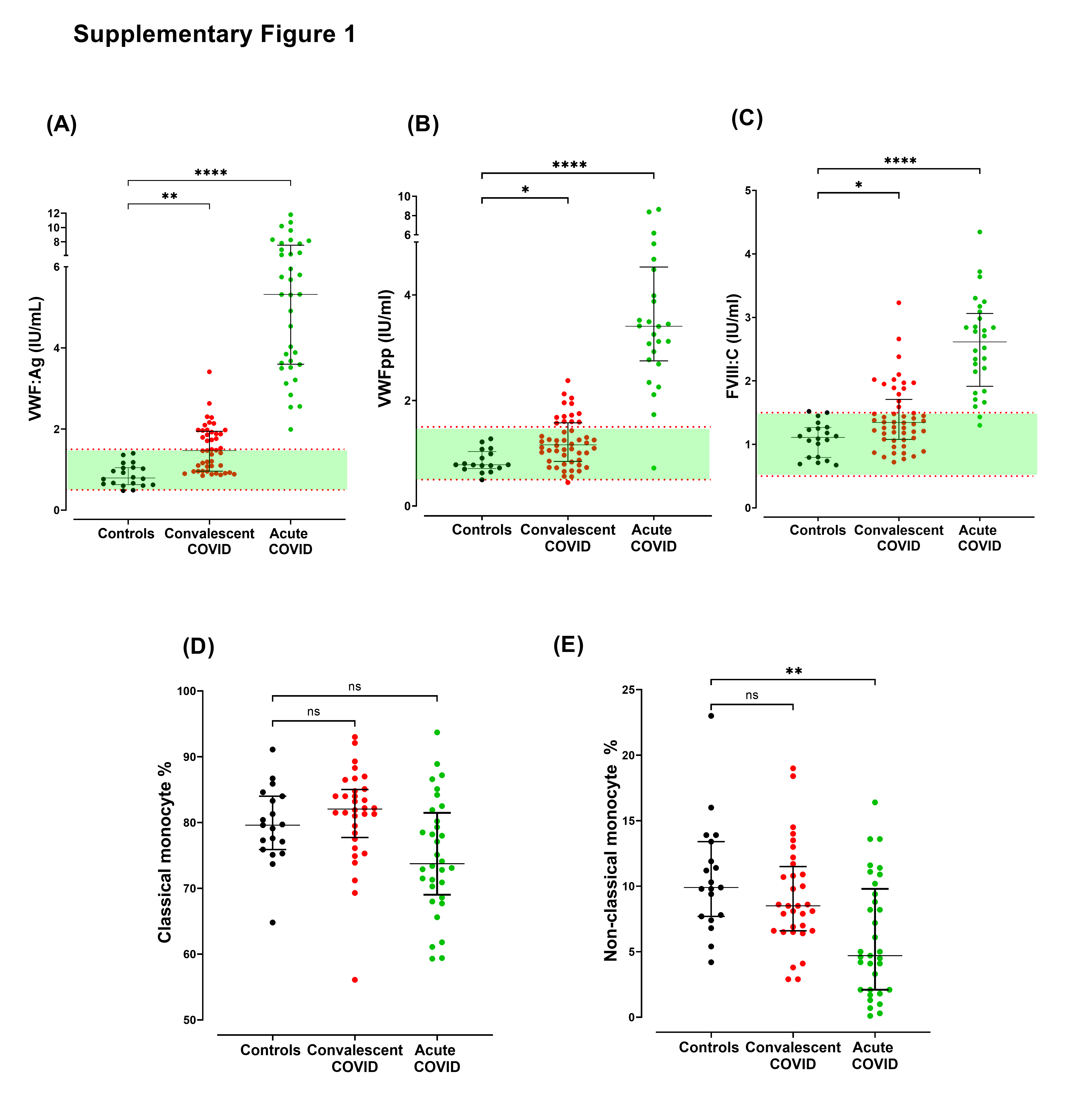

Supplement: Supplementary file 1 — Figure S1 [file JTH-20-2429-s003.tif]
