# Supplementary material for: Sustained VWF‐ADAMTS‐13 axis imbalance and endotheliopathy in long COVID syndrome is related to immune dysfunction
Source: J Thromb Haemost. 2022 Aug 4;20(10):2429–38. doi: 10.1111/jth.15830 (PMC9349977; doi:10.1111/jth.15830)
Supplement: Supplementary file 2 — Tables S1–S2 [file JTH-20-2429-s001.docx]

**Supplementary Table 1:**

|  | **Activated CD8 %** | | **Activated CD4 %** | | **Naïve CD4 %** | | **Naïve CD8 %** | | |
| --- | --- | --- | --- | --- | --- | --- | --- | --- | --- |
|  | **R^2^** | **P value** | **R^2^** | **P value** | **R^2^** | **P value** | **R^2^** | **P value** |  |
| **sTM** | 0.86 | <0.0001**** | 0.61 | 0.0002*** | -0.32 | 0.07 | -0.52 | 0.003** |  |
| **Ang-2** | 0.59 | 0.0004*** | 0.47 | 0.01* | -0.27 | 0.14 | -0.23 | 0.22 |  |
| **OPG** | 0.72 | <0.0001**** | 0.58 | 0.001** | -0.33 | 0.06 | -0.47 | 0.01* |  |
| **VWF:Ag** | 0.56 | 0.001** | 0.40 | 0.02* | -0.29 | 0.11 | -0.47 | 0.01* |  |
| **VWFpp** | 0.36 | 0.04* | 0.25 | 0.16 | 0.03 | 0.89 | -0.03 | 0.89 |  |
| **Factor VIII:C** | 0.38 | 0.04* | 0.22 | 0.24 | -0.16 | 0.40 | -0.38 | 0.04* |  |
| **VWF/ADAMTS13** | 0.51 | 0.003** | 0.41 | 0.02* | -0.30 | 0.10 | -0.48 | 0.01* |  |
| **D dimer** | 0.46 | 0.01* | 0.46 | 0.008** | -0.29 | 0.11 | -0.43 | 0.01* |  |
| **sCD25** | 0.61 | 0.0002*** | 0.61 | 0.0002*** | -0.11 | 0.56 | -0.30 | 0.92 |  |

|  | **Cell population** | **Beta-coefficient (95% CI)** | **P value** | **Cell population** | **Beta-coefficient (95% CI)** | **P value** |
| --- | --- | --- | --- | --- | --- | --- |
|  | **Activated CD8 %** |  |  | **Activated CD4 %** |  |  |
| **sTM** |  | 2.8 (1.8 – 3.7) | <0.0001**** |  | 0.26 (-0.1 – 0.6) | 0.11 |
| **Ang-2** |  | 0.004 (0.0001 – 0.01) | 0.02* |  | 0.001 (-0.001 – 0.01) | 0.17 |
| **OPG** |  | 0.01 (0.006 – 0.02) | <0.0001**** |  | 0.002 (-0.001 – 0.003) | 0.057 |
| **VWF:Ag** |  | 4.3 (-2.2 – 10.7) | 0.19 |  | 0.17 (-1.4 – 1.7) | 0.83 |
| **VWFpp** |  | 1.9 (-8.3 – 12.0) | 0.71 |  | -0.3 (-2.7 – 2.1) | 0.79 |
| **Factor VIII:C** |  | 4.0 (-2.9 – 1-.9) | 0.24 |  | 0.01 (-1.6 – 1.6) | 0.99 |
| **VWF/ADAMTS13** |  | 0.6 (-2.6 – 3.8) | 0.72 |  | -0.02 (-0.8 – 0.8) | 0.95 |
|  | **Naïve**  **CD8 %** |  |  | **Naïve**  **CD4 %** |  |  |
| **sTM** |  | -0.8 (-3.3 – 1.7) | 0.53 |  | -0.6 (-3.5 – 2.4) | 0.70 |
| **OPG** |  | -0.01 (-0.1 – 0.01) | 0.15 |  | 00.01 (-0.02 – 0.01) | 0.32 |
| **Ang-2** |  | 0.01 (-0.01 – 0.1) | 0.50 |  | -0.01 (-0.1 – 0.01) | 0.59 |
| **VWF:Ag** |  | -9.1 (-20.2 – 2.1) | 0.11 |  | -6.3 (-19.5 – 6.8) | 0.33 |
| **VWFpp** |  | 11.0 (-6.3 – 28.3) | 0.20 |  | 6.6 (-13.6 – 26.8) | 0.51 |
| **Factor VIII:C** |  | -9.6 (-21.3 – 2.0) | 0.10 |  | -1.3 (-12.8 – 10.2) | 0.81 |
| **VWF/ADAMTS13** |  | -2.4 (-8.0 – 3.1) | 0.38 |  | -1.7 (-8.1 – 4.7) | 0.59 |

**Supplementary Table 2:**
