# Supplementary material for: Sustained VWF‐ADAMTS‐13 axis imbalance and endotheliopathy in long COVID syndrome is related to immune dysfunction
Source: J Thromb Haemost. 2022 Aug 4;20(10):2429–38. doi: 10.1111/jth.15830 (PMC9349977; doi:10.1111/jth.15830)
Supplement: Supplementary file 3 — Data S1 [file JTH-20-2429-s002.docx]

**Supplementary Legends**

**Supplementary Figure 1:**

Comparison of plasma **(A)** VWF:Ag levels **(B)** VWFpp levels and **(C)** FVIII:C levels between patients with acute COVID-19 (n=36), convalescent COVID-19 (n=50) and healthy controls (n=20). **(D)** Classical and **(E)** Non-classical monocytes in peripheral blood were assessed using flow cytometry in convalescent COVID-19 patients (n=37), acute COVID-19 patients (n=32) and healthy controls (n=20). Data are presented as median and the interquartile range. Comparisons between groups were assessed by the Kruskal-Wallis test and Mann-Whitney U test as appropriate. (ns = not significant, *p<0.05, **p<0.01, ****p<0.0001).

**Supplementary Table 1:** Univariate regression analysis was performed comparing monocyte subsets with markers of endotheliopathy and coagulation activation**.**

**Supplementary Table 2:** Univariate regression analysis was performed comparing activated and naïve CD4+ and CD8+ T cells with markers of endotheliopathy and coagulation activation.
